# Contemporary Assessment and Management of Effusive-Constrictive Pericarditis

**DOI:** 10.1007/s11886-025-02326-4

**Published:** 2026-01-21

**Authors:** Lamis El Harake, Ashraf Samhan, Paul C. Cremer, Mohamed Al Kazaz

**Affiliations:** 1 Bluhm Cardiovascular Institute, Division of Cardiology, Chicago, IL USA; 2 Feinberg School of Medicine, Northwestern University, Chicago, IL USA; 3 Feinberg School of Medicine, Northwestern University, Bluhm Cardiovascular Institute, 675 N St Clair St Ste 19-100, Chicago, IL 60611 USA

**Keywords:** Effusive-constrictive pericarditis (ECP), Constrictive physiology (CP), Pericardial effusion (PE), Echocardiography (Echo), Pericardiectomy, Tuberculous pericarditis (TBP)

## Abstract

**Purpose of Review:**

Effusive-constrictive pericarditis (ECP) is a complex clinical condition that combines features of pericardial effusion/tamponade and constrictive pericarditis. The classic hemodynamic definition is persistent elevation of right atrial pressure despite drainage of a pericardial effusion. This review summarizes recent data on its epidemiology, pathophysiology, diagnosis, and management.

**Recent Findings:**

Prevalence varies from 2.4% to 14.8% depending on diagnostic criteria and etiology, reaching up to 50% in tuberculous pericarditis in endemic regions. Common causes include idiopathic, infectious (particularly tuberculous and bacterial), malignant, and post-surgical etiologies. While invasive hemodynamic assessment remains the reference standard, echocardiography is now the primary diagnostic tool, enabling recognition of constrictive physiology before and after pericardiocentesis. Cardiac magnetic resonance adds complementary information on pericardial thickness, inflammation, and potential for reversibility, aiding therapeutic decisions. Inflammatory ECP frequently resolves with medical therapy (NSAIDs, colchicine, corticosteroids, or IL-1 inhibitors) while tuberculous cases require antimicrobial therapy with corticosteroids in selected patients.

**Summary:**

ECP is a heterogeneous condition with variable clinical trajectories. Early identification through multimodality imaging is essential to guide therapy, target reversible inflammation, and prevent chronic constriction. Most inflammatory cases respond to anti-inflammatory treatment, whereas pericardiectomy is reserved for persistent, irreversible constrictive physiology despite optimal medical therapy.

**Supplementary Information:**

The online version contains supplementary material available at 10.1007/s11886-025-02326-4.

## Introduction

The pericardium is a double-walled sac surrounding the heart and the roots of the great vessels. It is invaginated by the heart and consists of fibrous and serous components. The serous pericardium is composed of a single layer of mesothelial cells with surface microvilli. These cells adhere directly to the heart, forming the visceral pericardium (or epicardium), and are contiguous with the parietal pericardium. The parietal pericardium refers to the combination of the outer dense fibrous layer and the parietal layer of the serosa pericardium [1, 2]. Acquired pericardial diseases commonly present as pericarditis, pericardial effusion, or constrictive pericarditis, each leading to varying degrees of hemodynamic compromise [3]. Chronic inflammation can lead to fibrosis and pericardial stiffening, resulting in impaired diastolic filling and symptoms of right-sided heart failure. Conversely, in pericardial effusion, accumulation of fluid under pressure can cause cardiac tamponade (CTP), a life-threatening condition caused by restricted cardiac filling [1, 4].

Although constrictive pericarditis and pericardial tamponade are distinct entities, clinical overlap may occur. Effusive–constrictive pericarditis represents a unique and often underrecognized syndrome that combines elements of both, characterized by the coexistence of a hemodynamically significant pericardial effusion and constrictive pathophysiology [5, 6]. First described through case reports in the 1950 s and formally defined by Hancock in 1971, effusive constrictive pericarditis is diagnosed by persistent elevation in right atrial pressure following pericardiocentesis, indicating continued visceral pericardial constriction and inflammation despite effusion drainage [5, 7]. Later studies refined the diagnostic criteria, defining effusive constrictive pericarditis as failure of the right atrial pressure to fall by at least 50% or to a level below 10 mmHg after normalization of intrapericardial pressure, assuming no severe tricuspid regurgitation or primary right ventricular failure [8]. This distinct hemodynamic entity, often associated with epicardial inflammation and a rigid visceral pericardium, is essential to identify. Misdiagnosis as isolated tamponade may delay appropriate treatment and prolong morbidity [7, 9].

Despite increasing awareness, ECP remains a challenging diagnosis. Even though only two prospective series by Sagristà-Sauleda and Ntsekhe have been published, ECP is gaining recognition as a distinct clinical entity, differing in both presentation and management from purely effusive or constrictive forms [8, 10]. Nevertheless, current understanding remains limited, as available data are primarily drawn from case reports, small series, and a few prospective studies. This review aims to provide a contemporary overview of effusive-constrictive pericarditis, focusing on its pathophysiologic mechanisms, diagnostic approach, and management with particular emphasis on multimodal imaging, invasive hemodynamics, and the role of medical and surgical therapies.

### Etiology and Epidemiology

Effusive constrictive pericarditis remains underdiagnosed, with its reported prevalence varying widely due to differences in diagnostic criteria and underlying etiologies. Early prospective studies using invasive hemodynamic monitoring identified ECP in 1.3% of patients with pericarditis and 6.8% of those presenting with tamponade [8]. Similarly, Nugue et al. reported ECP in 1.4% of 141 patients with pericardial effusion and 3.6% of those with chronic effusions, although invasive confirmation was lacking [11]. In a surgical series of 95 patients with confirmed constrictive pericarditis, ECP was found in 24%; notably, no cases were observed among postsurgical patients, who uniformly presented with dry fibrous constriction [9]. A more recent systematic review by Ntsekhe et al. of 642 patients systematically evaluated for ECP using imaging or invasive criteria. Reported prevalence ranged from 2.4% to 14.8%, with a pooled estimate of 4.5% [12]. The most common etiologies were idiopathic or viral (58%), followed by tuberculosis (38%), post-radiation (8%), and post-pericardiotomy (4%), as summarized in Table 1.

**Table 1:** Summary of reported etiologies in Effusive-Constrictive pericarditis

Study (Author, year)	Type of Study	Total ECP Patients (*N*)	Tuberculosis (%)	Idiopathic or post-infective (%)	Radiation (%)	Neoplastic (%)	Post-surgical (%)	Connective tissue (%)
Kim et al., 2018 [16]	Single center	33	0%	36.4%	0%	6.1%	18.2%	0%
Ntsekhe et al., 2012 [12]	Systematic review	26	38%	50%	8%	0%	4%	0%
Ntsekhe et al., 2013 [10]	Single center	36	100%	0%	0%	0%	0%	0%
Sagristá-Sauleda et al., 2004 [8]	Single center	15	7%	46.7%	13.3%	26.7%	7%	0%
Cameron et al., 1987 [9]	Single center	23	0%	39.1%	43.5%	4.3%	0%	13%

Geographic and socioeconomic factors influence the distribution of pericardial disease and, consequently, ECP. In high-income settings, over 80% of pericarditis cases are idiopathic or viral, with tuberculosis accounting for less than 5% [13]. In contrast, tuberculosis remains the leading cause of pericardial effusion in sub-Saharan Africa, responsible for up to 70% of cases [14]. Accordingly, ECP prevalence in tuberculous pericarditis cohorts ranges from 2.6% to 15%, though is often diagnosed without invasive confirmation [14, 15]. More definitive data come from a prospective study by Ntsekhe et al., who assessed 91 patients with suspected tuberculous pericarditis using combined pericardiocentesis and catheterization. Among the 68 individuals with confirmed disease, 52.9% had ECP, diagnosed by persistent elevation of right atrial pressure after fluid drainage [10]. These findings reinforce the importance of invasive assessment in regions where tuberculosis is endemic.

In a contemporary cohort of 205 patients undergoing pericardiocentesis, Kim et al. diagnosed ECP in 16% of patients using echocardiographic features such as respiratory variation in mitral inflow, septal shift, hepatic vein flow reversal, or annular reversus [16]. Pre-procedure tamponade and post-procedure hemopericardium were more common among those with ECP (52% vs. 36% and 33% vs. 13%, respectively). Etiologies included cardiac surgery (29%), idiopathic (25%), procedure-related (16%), and malignancy (12%). During follow-up, most patients improved with conservative management, with only two requiring pericardiectomy [16]. The high prevalence observed in the Kim et al. study raises questions about potential overdiagnosis when relying solely on echocardiographic criteria. As Klein and Cremer noted, evolving echocardiographic thresholds, particularly those emphasizing respiratory variation, may identify mild or transient forms of constrictive physiology. While this improves sensitivity, it may compromise specificity and introduce variability in reported rates [16, 17].

These studies collectively highlight the diagnostic complexity of ECP and its dependence on diagnostic methods and underlying disease burden. Standardized definitions and physiologic assessment remain essential for accurately characterizing its prevalence and natural history.

### Pathophysiology: Hemodynamics and the Role of Pericardial Inflammation

ECP is now recognized as a distinct clinical syndrome that combines the hemodynamic features of both pericardial tamponade and constrictive pericarditis [7]. In cardiac tamponade, intrapericardial pressure rises due to fluid accumulation, resulting in uniform external compression of the heart and impaired diastolic filling throughout the cardiac cycle [3, 18]. In contrast, constrictive pericarditis arises from pericardial fibrosis or inflammation, typically of the visceral layer, which restricts late diastolic expansion through a noncompliant pericardium despite normal or low pericardial pressure [1, 18]. ECP lies along a continuum between these two disorders and is defined by the persistence of elevated right atrial pressure after pericardiocentesis despite normalization of intrapericardial pressure [7, 19]. Patients may initially present with features of tamponade, but after drainage, signs of constrictive physiology emerge. Importantly, overt tamponade is not required. In the series by Ntsekhe et al., only 53% of patients with tuberculous pericardial effusions who met criteria for ECP had catheter-confirmed tamponade [10]. Similarly, Sagristà-Sauleda and colleagues found that, although all patients exhibited clinical tamponade and decreased right atrial pressure, the right atrial pressure contours did not match the classic tamponade physiology, emphasizing the unique hemodynamic pattern of EC [8]. Hancock further illustrated that right atrial waveforms in ECP have an intermediate relationship between the x and y descents when compared with tamponade and CP alone [7].

Beyond its hemodynamic definition, inflammation plays a central role in the pathophysiology of ECP [16]. Histologic and clinical observations demonstrate that active pericardial inflammation with edema, fibrin deposition, and inflammatory cell infiltration can transiently increase pericardial stiffness and impair diastolic filling even after fluid drainage [17]. Some cases resolve spontaneously. However, the degree and reversibility of this inflammatory response determines whether constriction can be transient and resolves with anti-inflammatory therapy or becomes refractory to medical therapy and progresses requiring radical pericardiectomy. Even in those cases that needs surgery, controlling inflammation before pursuing pericardiectomy is critical in lowering perioperative complications [2]. This inflammation-driven pathophysiology parallels the “inflammatory phenotype” described in acute or subacute pericarditis, characterized by elevated inflammatory markers, pericardial effusion and pericardial LGE with or without edema on T2-STIR imaging on CMR, which identifies patients who usually respond favorably to anti-inflammatory therapy [2, 20].

Anatomic and imaging findings further underscore this inflammatory substrate and illustrate the heterogeneity of ECP. In some cases, pericardial stiffness due to acute inflammation or edema may impair filling even in the absence of marked fibrotic thickening, though the prevalence of this presentation remains uncertain [21]. This supports the concept of transient constriction, a reversible form of constrictive pathophysiology. In one series, Sagristà-Sauleda et al. described patients with transient CP who improved with medical therapy alone, without requiring surgery [15]. These patients had inflammatory markers or late gadolinium enhancement on cardiac MRI, and effusion was present in most cases [22]. The mechanism involves a temporary loss of pericardial compliance due to the presence of fibrin and edema. Recognizing this potential for reversibility is crucial, as anti-inflammatory therapy (NSAIDs, colchicine, steroids, or IL-1 blockers) can restore normal physiology and avoid pericardiectomy [6, 18, 23].

Tuberculous pericarditis provides further insight into the immune mechanisms of ECP. Pericardial inflammation with intense fibrin deposition over the epicardium may cause temporary visceral constraint that reverses with antituberculous therapy in a substantial proportion of patients [24]. Inflammatory cytokines also likely play a pathogenic role. Ntsekhe et al. found that patients with tuberculous ECP had significantly higher serum and pericardial levels of interleukin 10 and interferon gamma compared to those with non-constrictive effusions, along with elevated transforming growth factor beta in the serum [10]. These mediators may promote fibrosis or prolong inflammation, contributing to ECP development.

In non-tuberculous ECP, florid inflammation also appears to play a role. Kim et al. observed that although the total pericardial white blood cell count was similar in patients with and without ECP, the percentage of neutrophils was higher in the ECP group, suggesting a more intense inflammatory process [16]. Their study also showed that constrictive features on echocardiography can aid in diagnosing residual constriction after pericardiocentesis. Notably, most patients with ECP in that series did not require surgery, and constriction resolved spontaneously or with medical therapy in the majority [16].

These findings collectively highlight the intricate and heterogeneous characteristics of ECP. It is not merely an intermediate stage between tamponade and CP, but rather a clinical spectrum influenced by distinct factors such as pericardial fluid, inflammation, fibrosis, and compliance, as summarized in Table 2. This variability in underlying pathophysiology accounts for the diverse clinical presentations and diagnostic challenges, as well as the differing courses observed in treatment management.

**Table 2:** Comparative features of pericardial compressive syndromes.

	Constrictive Pericarditis	Effusive Constrictive Pericarditis	Cardiac Tamponade
Pathophysiology	Chronic inflammation that leads to fibrosis, thickening, and pericardial stiffening **→** impaired early diastolic filling; abrupt cessation of filling mid-diastole due to non-compliant pericardium.	Combination of pericardial effusion and constrictive visceral pericardium	Accumulation of fluid under pressure in the pericardial sac that restricts cardiac filling **→ **external compression of the heart, primarily restricting late diastolic filling.
Key hemodynamics	• Impaired ventricular filling due to large fluid volume or rigid, scarred pericardium • Prominent y descent • Equalization of diastolic pressures in all four chambers • ‘Dip-and-plateau’ pattern in RV pressure tracings	• Persistent ↑ in RAP after pericardial effusion drainage • Prominent y descent • Failure of RAP to fall < 10 mmHg or by 50% after pericardiocentesis • ‘Dip-and-plateau’ pattern on RV pressure tracings	• ↓ CO due to compression of the heart secondary to ↑ intrapericardial pressure • Blunted y descent • Elevated and equalized diastolic pressures in all four chambers

*RAP* right atrial pressure, *RV* right ventricle, *CO* cardiac output

### Diagnosis

#### Clinical Presentation and Physical Exam

Due to its mixed pathophysiology, ECP presents with a wide range of clinical features, complicating timely diagnosis [8]. Patients may initially exhibit acute signs of tamponade such as hypotension, tachycardia, elevated jugular venous pressure (JVP), and pulsus paradoxus, or sub-acutely with symptoms more typical of constriction, including fatigue, exertional dyspnea, peripheral edema, hepatomegaly, and ascites [8, 16, 19]. Chest pain, low-grade fever, and a pericardial friction rub may indicate active pericardial inflammation [10, 25, 26]. A key clinical clue is the persistent elevation of JVP after pericardiocentesis, once pericardial pressure has normalized and other causes such as tricuspid regurgitation are excluded [18, 21]. The jugular venous waveform may demonstrate prominent x and y descents, similar to constrictive physiology [7]. In Kim et al.’s cohort, ECP was diagnosed post-procedure in 16% of patients undergoing pericardiocentesis, with a higher rate of pre-procedure hemodynamic instability and effusive disease [16].

In this setting, laboratory markers can support clinical evaluation by identifying ongoing inflammation and potential reversibility. In Feng et al.’s study, 76% of patients with reversible constrictive pericarditis, identified by clinical signs and elevated inflammatory markers, were successfully managed with medical therapy alone, without requiring surgery [22]. This underscores the prognostic value of inflammatory markers such as CRP and ESR in identifying patients likely to respond to anti-inflammatory treatment. CRP is also used to guide tapering of anti-inflammatory therapy, while ESR and troponin may aid in identifying systemic inflammation or myocardial involvement [27]. As previously discussed, immune-mediated pathways, including cytokine activity seen in tuberculous ECP, may further contribute to disease persistence and constriction [10].

#### Echocardiography

Echocardiography is the first line and cornerstone modality for diagnosing ECP, as it enables the assessment of hemodynamic changes both before and after pericardiocentesis [28, 29]. Findings typically fall between those seen in tamponade and constrictive pericarditis, reflecting the syndrome’s mixed physiology. When paired with Doppler and respiratory monitoring, echocardiography also plays a key role in identifying constrictive pathophysiology across the broader pericardial disease spectrum, including constrictive pericarditis and tamponade. Features such as diastolic chamber collapse, septal shift, inferior vena cava dilation, and pericardial adhesions are more reliably detected using a comprehensive protocol that incorporates M-mode, tissue Doppler, hepatic vein Doppler, and, when available, 3D imaging [1].

The diagnostic utility of echo-Doppler for ECP was initially evaluated in a cohort of 32 patients with tuberculous pericarditis undergoing pericardiocentesis. Using invasive hemodynamic criteria as the gold standard, they found that echocardiographic signs, specifically ≥ 25% respiratory variation in the mitral E wave or respirophasic septal shift, had a sensitivity of 81% and specificity of 75% for diagnosing EC [30]. Tissue Doppler and hepatic vein Doppler were not assessed. More comprehensive data have since emerged. In a study of 205 consecutive patients undergoing pericardiocentesis, Kim et al. aimed to characterize the incidence and diagnostic echocardiographic findings of ECP [16]. Patients with ECP more frequently exhibited respirophasic septal shift (21% vs. 1%), mitral inflow variation (89% vs. 62%), late diastolic hepatic vein flow reversal (48% vs. 23%), and higher medial e′ velocities (8.9 vs. 6.9 cm/s) compared to those with isolated effusive pericarditis. However, as Klein and Cremer noted in their editorial, this study relied on earlier Doppler criteria and did not incorporate the updated Mayo Clinic tissue velocity thresholds. Moreover, systematic post-drainage evaluation was lacking, limiting the ability to detect constrictive pathophysiology that may only become evident once pericardial pressure normalizes [17].

After pericardiocentesis, constrictive pathophysiology typically becomes more apparent. Findings include persistent right atrial pressure elevation, unchanged mitral E velocities, persistent hepatic vein diastolic flow reversal, and near-universal presence of respirophasic septal shift, mitral inflow variation, and IVC dilation [16]. Miranda et al. similarly noted that while medial and lateral e′ velocities were higher in ECP than in tamponade, hepatic vein diastolic flow remained reduced in both groups. Respiratory variation in transmitral E: A ratios was greater in ECP than in tamponade but less than in CP [29].

Together, these findings support the use of post-drainage echocardiography protocols for ECP evaluation, as reinforced by the 2024 international consensus statement, which recommends a comprehensive echocardiographic approach for assessing tamponade, CP, and ECP [1].

#### Multimodality Imaging

Cardiac magnetic resonance imaging (CMR) and computed tomography (CT) are valuable complementary tools for evaluating ECP. CT can assess pericardial thickening, effusion size and distribution, fluid characteristics, calcifications, and contrast enhancement, features that may suggest underlying inflammation. CMR provides high-resolution tissue characterization and can detect active pericardial inflammation through late gadolinium enhancement (LGE), which reflects neovascularization and fibroblast activity; in the appropriate clinical context, this may indicate inflammation [22, 31]. This can help identify patients with potentially reversible constriction who may benefit from anti-inflammatory therapy.

In ECP, CMR and CT often reveal a pericardial effusion accompanied by pericardial thickening or enhancement, typically evident before drainage. These modalities are beneficial in detecting complex or loculated effusions, which are common in tuberculous or postsurgical cases [32, 33]. Both modalities are valuable for evaluating structural and inflammatory features. CMR also assesses myocardial involvement through tissue mapping and LGE, while CT is particularly useful for identifying calcifications and aiding in surgical planning. Together, they complement Doppler echocardiography in assessing suspected CP or ECP [1, 18].

F-18 fluorodeoxyglucose positron emission tomography (FDG-PET) may also have a role in identifying reversible inflammation, although its application in ECP remains unclear [32, 34]. In a cohort of 16 patients with CP, including those with tuberculous cases, FDG-PET accurately predicted symptom resolution and improvement in constrictive pathophysiology following corticosteroid therapy [34].

#### Cardiac Catheterization

**Fig. 1:**
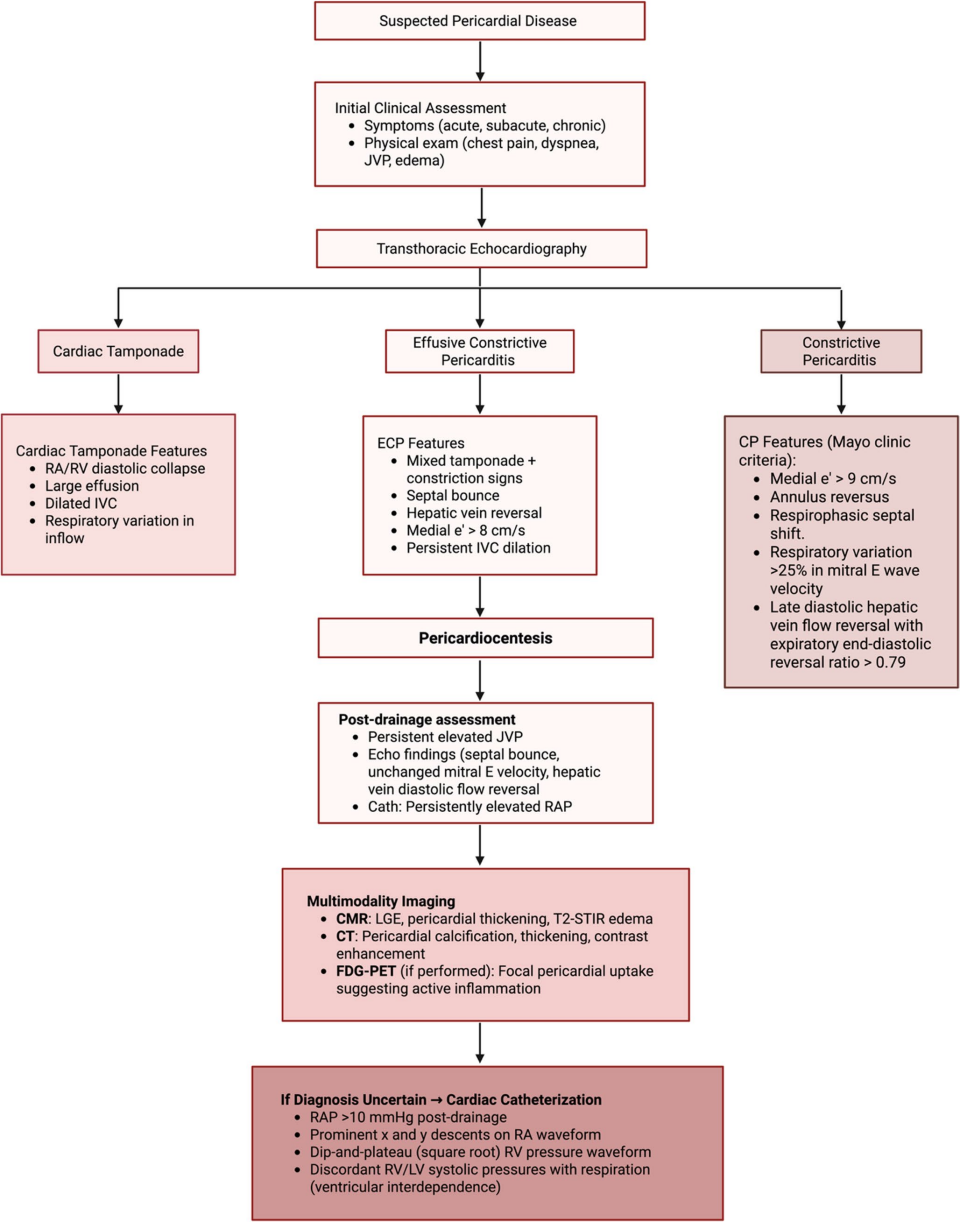
Diagnostic Algorithm for Effusive-Constrictive Pericarditis. Diagnostic algorithm for effusive–constrictive pericarditis (ECP). All patients with suspected pericardial disease undergo clinical assessment followed by transthoracic echocardiography (TTE). Echocardiographic findings classify patients into tamponade, ECP, or constrictive pericarditis (CP). In ECP, pericardiocentesis and post-drainage Doppler evaluation confirm persistent constrictive physiology. Multimodality imaging (CMR, CT, FDG-PET) further characterizes pericardial anatomy and inflammation. In cases with diagnostic uncertainty, invasive hemodynamic assessment via cardiac catheterization is performed. (Created in BioRender. Harake, L. (2025) https://BioRender.com/agn1lgt.) Key: ECP: effusive–constrictive pericarditis; CP: constrictive pericarditis; RA/RV: right atrium/right ventricle; IVC: inferior vena cava; e′: early diastolic mitral annular velocity; Cath: cardiac catheterization; Right atrial pressure (RAP); CMR: cardiac magnetic resonance; LGE: late gadolinium enhancement; CT: computed tomography; FDG-PET: fluorodeoxyglucose positron emission tomography; RV/LV: right ventricle/left ventricle

Cardiac catheterization is not routinely required to diagnose ECP, but it remains valuable in ambiguous cases. Historically, invasive hemodynamics were central to diagnosis [7]. Sagristà-Sauleda et al. proposed diagnostic criteria including a right atrial pressure that fails to fall below 10 mmHg or by at least 50% following pericardiocentesis [8]. Before drainage, catheterization may reveal intermediate right atrial pressure waveforms, including persistent V waves and x and y descents, features that fall between those of tamponade and constrictive pericarditis [8, 21]. After drainage, persistent elevation in right atrial pressure, absent inspiratory decline, prominent y descent, and a dip-and-plateau configuration in right ventricular pressure tracings are supportive of ECP [7, 21]. These hemodynamic findings must be interpreted in clinical context, as elevated right atrial pressure may also result from right heart failure or severe tricuspid regurgitation. In addition, simultaneous right and left heart catheterization may be helpful. Findings such as discordant respirophasic changes in ventricular systolic pressures and a systolic area index greater than 1.1 reflect exaggerated ventricular interdependence and are highly suggestive of constrictive physiology [27]. The proposed diagnostic approach for ECP, incorporating clinical, imaging, and hemodynamic assessment, is outlined in Fig. 1.

### Treatment and Prognosis

#### Medical Therapy

ECP can be transient and that is a key distinction from chronic constrictive pericarditis that is characterized by irreversible pericardial thickening and fibrosis [1]. ECP can be as result of inflammatory pericarditis and targeting that can reverse the pathophysiology. Chronic constrictive pericarditis usually requires surgical radical pericardiectomy [2]. Management of effusive-constrictive pericarditis centers on addressing the underlying etiology, reducing inflammation, and alleviating constrictive pathophysiology. In patients with tuberculous pericarditis, appropriate antimicrobial therapy is essential [35, 36]. Evidence on the role of adjunctive corticosteroids is mixed [37–39]. An earlier randomized trial in Transkei demonstrated that adding corticosteroids to standard anti-tuberculous therapy in patients with constrictive tuberculous pericarditis accelerated clinical recovery and reduced the subsequent need for pericardiectomy [37]. However, the larger Investigation of the Management of Pericarditis in Africa (IMPI) trial, which enrolled 1400 patients with definite or probable tuberculous pericarditis, found no mortality benefit from routine corticosteroid use. Although prednisolone reduced the incidence of constrictive pericarditis compared with placebo, it was associated with a higher risk of HIV-associated malignancies, particularly Kaposi sarcoma [39]. These findings suggest that corticosteroids should not be used routinely in all patients with tuberculous pericarditis but may be considered in selected high-risk cases, particularly in those without HIV infection who present with evidence of constriction.

In idiopathic or post-surgical cases, management parallels that of transient constrictive pericarditis. Inflammation may be suggested by elevated inflammatory markers (e.g., CRP), pericardial effusion, and imaging features such as LGE or T2 signal intensity on CMR, all of which may indicate potentially reversible disease [1, 18]. ECP might resolve spontaneously or with anti-inflammatory therapy given for 3–6 months. However, this needs close clinical monitoring with inflammatory biomarker, serial echocardiograms and CMRs. If refractory after 3–6 months or worsening sooner, then surgical intervention is pursued after inflammation is controlled [40].

**Fig. 2:**
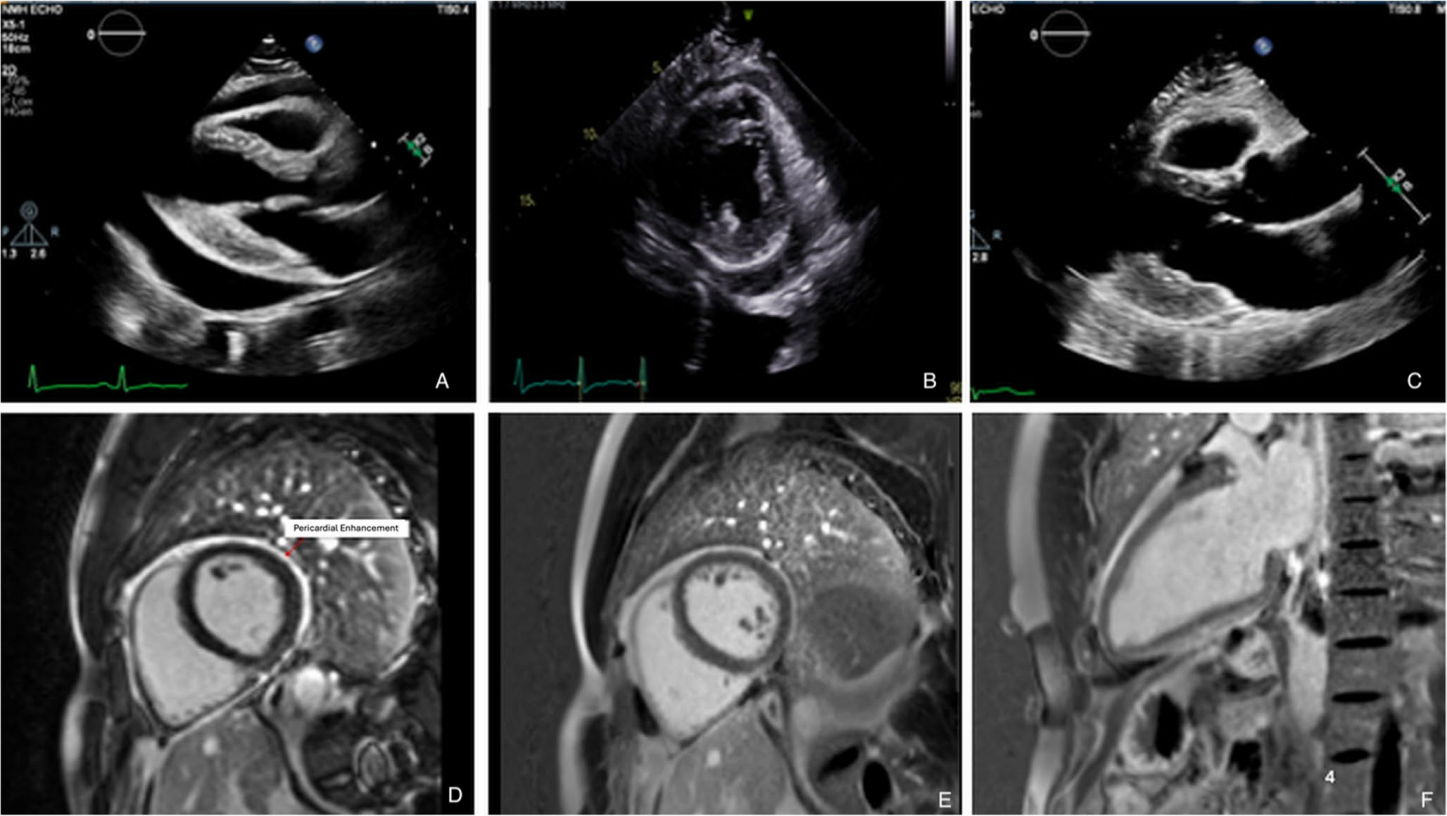
AU: Case example of effusive–constrictive pericarditis with resolution after anti-inflammatory therapy and Rilonacept. Serial transthoracic echocardiography videos demonstrate **A** pericardial effusion, **B** persistent constrictive physiology after pericardiocentesis, and **C** resolution following treatment with NSAIDs, colchicine, prednisone, and subsequent Rilonacept monotherapy. Cardiac magnetic resonance (CMR) images show**D** pericardial late gadolinium enhancement (LGE) and **E, F** resolution of pericardial inflammation after 18 months of Rilonacept therapy

Initial treatment generally includes nonsteroidal anti-inflammatory drugs (NSAIDs) and colchicine [19]. NSAIDS may be contraindicated in patients with heart failure, peptic ulcer disease or renal failure. In the patients with heart failure or significant symptoms, addition of corticosteroids is reasonable [2]. In cases of persistent inflammation or inadequate response, escalation to corticosteroids or IL-1 inhibitors (e.g., anakinra or rilonacept) may be considered, though randomized trials comparing these strategies are lacking [2, 31, 40]. A representative case of transient ECP with resolution following anti-inflammatory therapy and Rilonacept is shown in Fig. 2, demonstrated by transthoracic echocardiography videos (A–C) and corresponding CMR images (D–F). Overall, therapy is often continued for months, or even years, tailored to the trajectory of clinical and imaging findings.

#### Pericardial Effusion Drainage

In acute presentations with hemodynamic compromise due to cardiac tamponade, pericardiocentesis remains the initial, time sensitive therapeutic step and often leads to symptomatic improvement [7, 8]. Surgical pericardial windows are typically pursued for when there is no safe percutaneous option or in cases of recurrent effusion, which is common in malignant etiologies. However, inflammatory or idiopathic ECP may exhibit transient constrictive physiology that resolves with medical therapy alone, as suggested by small series, and pericardial windows are generally avoided in these cases to prevent additional pericardial injury or exacerbation of inflammation [6, 14, 37]. Progression to chronic constriction may still occur, warranting close follow-up [7]. In the study by Kim et al., only 2 of 33 patients with ECP progressed to persistent constriction on follow-up echocardiography [16]. However, the true risk of progression remains unclear, as current data are limited to observational findings from small cohorts.

#### Surgical Management

Pericardiectomy is reserved for patients with refractory symptoms who have failed or are intolerant to medical therapy, or for those with chronic, irreversible constrictive pericarditis. Surgical intervention is most appropriate in cases of persistent constrictive physiology accompanied by New York Heart Association (NYHA) class III–IV symptoms, recurrent pericardial effusions, or refractory recurrent pericarditis [2, 21, 40]. In ECP, constriction often arises from the visceral pericardium, making complete resection challenging [21]. Complete resection may not be feasible due to dense epicardial inflammation and fibrosis involving both visceral and parietal layers. In such cases, a checkerboard or waffle technique consisting of multiple longitudinal and transverse incisions across the epicardial rind can facilitate diastolic expansion without full excision, though this approach can be avoided with adequate anti-inflammatory treatment prior to surgery [40–42]. Notably, regions where the parietal pericardium has previously been resected, such as following a pericardial window, often demonstrate less epicardial inflammation during subsequent surgery.

### Prognosis

The prognosis of ECP is heterogeneous ranging from resolution with medical treatment to progression to dry constrictive pericarditis. In the review of 20 patients, Ntsekhe et al. reported a 65% pericardiectomy rate, with higher rates in idiopathic ECP (73%) compared to tuberculous (60%) or other etiologies (50%) [12]. In contrast, the single-center study by Kim et al. found that only 2 of 33 patients required surgery over nearly four years of follow-up, suggesting that early recognition and timely anti-inflammatory treatment may improve outcomes in selected patients [16]. Mortality rates after pericardiectomy for chronic constrictive pericarditis range from 4% to 8% [41–43]. However, it is important to recognize that risk is more strongly influenced by patient substrate than by the procedure itself. For example, surgical outcomes are significantly worse in patients with prior radiation exposure or advanced comorbidities compared to younger, otherwise healthy individuals with idiopathic disease.

In tuberculous ECP, outcomes are influenced by coinfections and treatment response. In the systematic review by Ntsekhe et al., the pericardiectomy rate was approximately 60% in TB-related cases, and mortality was frequently driven by comorbid conditions such as HIV co-infection [12]. The IMPI trial reported that the composite outcome of death, cardiac tamponade requiring pericardiocentesis, or constrictive pericarditis occurred in approximately 24% of patients, with no significant difference between treatment and placebo groups [39]. Despite appropriate anti-tuberculous therapy, disease progression may occur, highlighting the need for close follow-up.

#### Individualized Approach

**Fig. 3:**
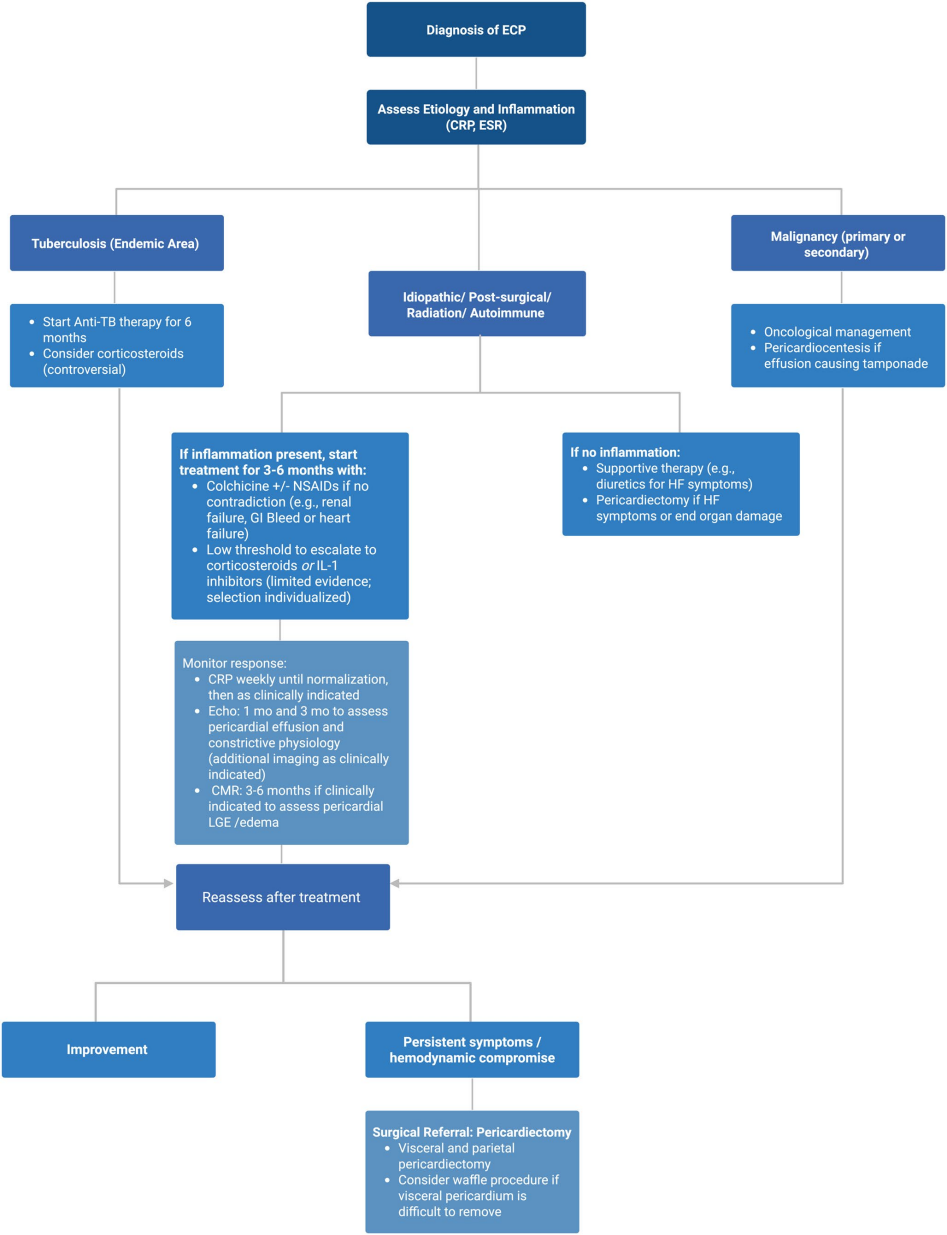
Summary of Management Approach to Effusive-Constrictive Pericarditis. Treatment is guided by underlying etiology and inflammatory status. Tuberculous pericarditis (in endemic areas) is managed with 4-drug antituberculosis therapy for 6 months; adjunctive corticosteroids may be considered. Malignant effusions require oncologic therapy and pericardiocentesis if tamponade is present. In idiopathic, post-surgical, radiation-induced, or autoimmune cases, active inflammation warrants NSAIDs plus colchicine; refractory cases may receive corticosteroids or anakinra (limited evidence). In the absence of inflammation, conservative monitoring is recommended. Patients with persistent symptoms or hemodynamic compromise should undergo pericardiectomy, with the waffle procedure considered if visceral pericardium is difficult to remove. (Created in BioRender. Harake, L. (2025) https://BioRender.com/zt4teh0.) CRP: C-reactive protein; ESR: erythrocyte sedimentation rate; CMR: cardiac magnetic resonance; LGE: late gadolinium enhancement; HF: heart failure; NSAIDs: nonsteroidal anti-inflammatory drugs; IL-1: interleukin-1; f/u: follow-up; ECP: effusive-constrictive pericarditis; mo: months

Management decisions should be individualized, based on the underlying etiology, symptom severity, and imaging findings. Patients with inflammatory forms may respond to medical therapy, while those with persistent symptoms or evidence of irreversible constriction may ultimately require surgery. In the absence of standardized guidelines, current management strategies are primarily informed by expert consensus and small case series [18, 19]. The proposed management approach for ECP, incorporating medical, procedural, and surgical strategies, is illustrated in Fig. 3.

#### Future Directions

Despite growing recognition of ECP as a distinct entity on the pericardial disease spectrum, several knowledge gaps persist. Future studies are needed to determine the diagnostic accuracy of non-invasive imaging modalities, mainly echocardiography and CMR, relative to invasive hemodynamic assessment, and to establish standardized criteria for identifying ECP across various etiologies. Moreover, reports suggesting that the visceral pericardium or the effusion itself may have distinct imaging features require systematic validation [44]. The role of serum or pericardial biomarkers reflecting inflammation or fibrosis in diagnosis and monitoring also remains incompletely validated [10].

From a therapeutic standpoint, decision-making continues to be largely empiric. There is limited evidence guiding the optimal duration and tapering of anti-inflammatory therapy, criteria for escalation of care, or timing of surgical referral. Further research is needed to identify predictors of response to medical therapy and progression to chronic constriction, which may help refine individualized management strategies. Importantly, comparative studies examining the upfront use of corticosteroids versus IL-1 blockers are lacking, despite increasing off-label use of IL-1 inhibitors in refractory cases [45]. Emerging therapies targeting the NLRP3 inflammasome are currently under investigation and may expand future treatment options for patients with recurrent or treatment-resistant pericarditis [46]. 

## Conclusions

Effusive-constrictive pericarditis is a unique overlap of tamponade and constrictive pathophysiology, often occurring in cases of pericardial inflammation. The diagnosis requires a high level of suspicion, especially when elevated filling pressures remain after pericardiocentesis. While echocardiography is central to recognition, no single test is definitive, and a multimodal imaging approach is often necessary. Treatment should be based on the underlying cause, with anti-inflammatory therapy as the first-line treatment for inflammatory cases and pericardiectomy reserved for refractory disease. Clinicians should remain vigilant for progression to chronic constriction and closely reassess patients after initial therapy. As diagnostic criteria improve and awareness grows, early detection and personalized management will be essential for better outcomes.

## Key References

- Klein AL, Wang TKM, Cremer PC, et al. Pericardial Diseases. JACC: Cardiovascular Imaging. 2024;17(8):937–988. 10.1016/j.jcmg.2024.04.010.
  This review provides an up-to-date overview of pericardial diseases, highlighting diagnostic advances with multimodality imaging and evolving medical and surgical management strategies.
- Sagristà-Sauleda J, Angel J, Sánchez A, Permanyer-Miralda G, Soler-Soler J. Effusive-constrictive pericarditis. N Engl J Med. 2004;350(5):469–475. 10.1056/NEJMoa035630.
  In a prospective series of 15 patients among 190 undergoing combined pericardiocentesis and catheterization for tamponade, persistent constrictive physiology was frequent and often required visceral pericardiectomy, though some idiopathic cases resolved spontaneously.
- Kim KH, Miranda WR, Sinak LJ, et al. Effusive-Constrictive Pericarditis After Pericardiocentesis: Incidence, Associated Findings, and Natural History. JACC Cardiovasc Imaging. 2018;11(4):534–541. 10.1016/j.jcmg.2017.06.017.
  Evaluated the incidence and course of effusive–constrictive pericarditis following pericardiocentesis, finding that it occurs more frequently than previously recognized and often has distinct fluid and imaging characteristics that can guide early diagnosis and prognosis.
- Klein AL, Cremer PC. Ephemeral Effusive Constrictive Pathophysiology∗. JACC: Cardiovascular Imaging. 2018;11(4):542–545. doi:10.1016/j.jcmg.2017.10.028.
  Discussed the concept of transient effusive–constrictive pathophysiology, emphasizing the importance of recognizing potentially reversible cases and tailoring management based on close follow-up and multimodality imaging rather than immediate surgery.
- Ntsekhe M, Shey Wiysonge C, Commerford PJ, Mayosi BM. The prevalence and outcome of effusive constrictive pericarditis: a systematic review of the literature. Cardiovasc J Afr. 2012;23(5):281–285. doi:10.5830/CVJA-2011-072.
  Randomized trial showing that neither prednisolone nor M. indicus pranii reduced a composite of death, tamponade, or constriction in tuberculous pericarditis, though prednisolone reduced constriction and hospitalization rates.
- Al-Kazaz M, Klein AL, Oh JK, et al. Pericardial Diseases and Best Practices for Pericardiectomy. Journal of the American College of Cardiology. 2024;84(6):561–580. doi:10.1016/j.jacc.2024.05.048.
  State-of-the-art review of pericardial diseases with an emphasis on pericardiectomy, recommending radical resection at high-volume centers for chronic or refractory constrictive pericarditis, and outlining a multidisciplinary approach from diagnosis to recovery.

## Supplementary Information

Below is the link to the electronic supplementary material.

ESM 1 MOV (6.71 MB)

ESM 2 MOV (11.8 MB)

ESM 3 MOV (13.2 MB)

## Data Availability

This article does not contain any original data. All data discussed are available in the cited published literature.

## Abbreviations

ECP: Effusive-constrictive pericarditis
pEff: Pericardial effusion
CP: Constrictive pericarditis
CT: Computed tomography
CTP: Cardiac tamponade
CMR: Cardiac magnetic resonance
NSAIDs: Non-steroidal anti-inflammatory drugs
JVP: Jugular venous pressure
IVC: Inferior vena cava
LGE: Late gadolinium enhancement
